# Pyroptosis executive protein GSDMD as a biomarker for diagnosis and identification of Alzheimer’s disease

**DOI:** 10.1002/brb3.2063

**Published:** 2021-02-15

**Authors:** Heping Shen, Chenyang Han, Yi Yang, Li Guo, Yongjia Sheng, Jin Wang, Wenyan Li, Liping Zhai, Genghuan Wang, Qiaobing Guan

**Affiliations:** ^1^ Department of Neurology The Second Affiliated Hospital of Jiaxing University Jiaxing China; ^2^ Department of Pharmacy The Second Affiliated Hospital of Jiaxing University Jiaxing China; ^3^ Department of Center Laboratory The Second Affiliated Hospital of Jiaxing University Jiaxing China

**Keywords:** Alzheimer's disease, diagnosis, GSDMD, identification, pyroptosis

## Abstract

**Objective:**

This study was mainly conducted to explore the expression changes of GSDMD and conventional markers (including T‐Tau, Tau181p, and Aβ_1–42_) in the cerebrospinal fluid among patients with Alzheimer's disease (AD) and vascular dementia (VD), followed by determination of role of GSDMD in diagnosing and identifying AD and VD.

**Methods:**

In this study, 60 patients with VD, 60 patients with AD, and 50 healthy controls were enrolled. Lumbar puncture was performed to collect cerebrospinal fluid samples. Patients with VD and patients with AD were evaluated using the Mini‐Mental State Examination (MMSE) scale, Montreal Cognitive Assessment (MoCA) scale, Clinical Dementia Rating (CDR) scale, Activity of Daily Living (ADL) scale, and Neuropsychiatric Inventory (NPI) questionnaire, aiming to determine the behavioral ability of patients. ELISA kit was purchased to determine the levels of GSDMD, T‐Tau, Tau181p, and Aβ_1–42_ in cerebrospinal fluid, and the expression of inflammatory factors, IL‐1β and IL‐6, was also detected.

**Results:**

(1) The levels of GSDMD, T‐Tau, and Tau181p in the cerebrospinal fluid were higher in patients with AD than those of patients with VD and healthy controls, while the levels of Aβ_1‐42_ in the cerebrospinal fluid were lower in patients with AD than that in healthy controls and patients with VD. (2) GSDMD had good diagnostic accuracy in AD. Additionally, GSDMD, T‐Tau, Tau181p, and Aβ_1‐42_ had good discrimination accuracy in distinguishing AD and VD. (3) The expression levels of inflammatory factors (IL‐1β and IL‐6) in cerebrospinal fluid were higher in patients with AD than those of healthy controls and patients with VD, which were positively correlated with GSDMD expression.

**Conclusion:**

The expression of GSDMD was increased in patients with AD, which could be used as a biomarker for AD diagnosis and identification from VD.

## Graphical abstract

The expression of GSDMD was increased in AD patients, which could be used as a biomarker for AD diagnosis and identification from VD.

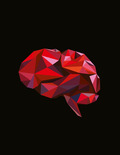

## 1 BACKGROUND

Dementia is currently a global public health problem. There have been over 35 million patients with dementia worldwide by 2010, with an estimated 100 million patients with dementia by 2050 (Reynish et al., 2017; Tan et al., 2018). Alzheimer's disease (AD) is a common central degenerative disorder, accompanied by neurological dysfunction such as dementia and abnormal behavior. And AD is also one of the biggest causes of dementia (Robinson et al., 2017). Vascular dementia (VD) is another high‐incidence cognitive disorder. Generally, patients are burdened with secondary dementia due to cerebrovascular disease, commonly including post‐stroke dementia, cerebrovascular dementia, and frontal lobe dementia (Smith, 2017). From the pathological perspective, the pathogenesis of AD and VD is different, while they are commonly manifested as cognitive impairment. At present, there is no reliable method for the diagnosis of two diseases in the clinical practice. Biomarkers are quantitative measurements that reflect the dynamic changes of progressive diseases, which can be used as the objective diagnostic and therapeutic basis. There are rarely any reports of biomarkers for AD and VD; however, the identification between AD and VD is of great significance. The cerebrospinal fluid communicates with brain cells and the extracellular environment, which can more authentically reflect the pathology of brain tissue more than peripheral blood. Previous studies have found that the expression levels of Aβ and Tau in AD can be used as diagnostic markers for AD (Herukka et al., 2017; Humpel & Hochstrasser, 2017). Aβ is an amyloid protein, and the excessive deposition of Aβ in brain tissue can induce the formation of senile plaques, which is also an important pathological characteristic of AD. Tau is a tubulin and can form neurofibrillary tangles (NFTs) after phosphorylation. p‐Tau (Tau181p) and Aβ_1–42_ are the main toxic substances (Agostino et al., 2015). In the diagnostic criteria of AD, including 2007 National Institute of Neurological and Communicative Disorders and Stroke‐Alzheimer's Disease and Relate Disorders Association (NNINCDS‐ADRDA) and 2011 revised National Institute on Aging‐Alzheimer's Association (NIA‐AA), Aβ_1–42_ and tau protein are emphasized as AD characteristic pathological biomarkers and diagnostic tools (Knopman et al., 2018; Mathuranath et al., 2012), suggesting that Aβ_1–42_ and Tau are the current gold standards for AD diagnosis.

Pyroptosis is a new type of inflammatory cell death, mainly depending on caspase family. To be specific, caspase‐1 can mediate the cleavage of downstream gasdermin D (GSDMD) and pro‐IL‐1β (Gutierrez et al., 2017). After cleavage of GSDMD into p30‐GSDMD, the p30‐GSDMD protein is oligomerized to form an oligomer, to further anchored on the cell membrane, resulting in the formation of cell membrane pores, thereby increasing cell osmotic pressure, causing membrane rupture and release of massive inflammatory factors (Kambara et al., 2018). Therefore, GSDMD is called the executive protein of pyroptosis. At present, several studies have demonstrated that pyroptosis plays an important role in mediating the occurrence of neuroinflammation. Inflammation has been clearly reported to be involved throughout the entire occurrence and progression of AD in terms of the pathological mechanism of AD. A large number of studies have also shown that neuroinflammation inhibition can regulate the cognitive function of AD (Chen et al., 2016). GSDMD, as an executive protein of pyroptosis, plays an important role in pyroptosis‐mediated neuroinflammation, which is also a relatively sensitive protein. Therefore, it is speculated that GSDMD may indicate AD, which might even be used as an indicator to identify AD from VD.

In this study, ELISA was used to detect the levels of GSDMD, T‐Tau, Tau181p, and Aβ_1‐42_ in the cerebrospinal fluid of patients with AD, patients with VD, and controls (Control). Meanwhile, the levels of inflammatory factors, including IL‐1β and IL‐6, were also assessed. These indicators were explored whether they could be used as a marker for AD diagnosis or VD identification, thereby providing novel references for the study of AD biomarkers.

## 2 MATERIAL AND METHODS

### 2.1 Case source and criteria

A total of 170 subjects were enrolled in this study, including 60 patients with AD, 60 patients with VD and 50 controls. Among the 60 patients with AD, there were 28 males and 32 females, aged 53–83 years old. Among the 60 patients with VD, there were 31 males and 29 females, aged 50–86 years old. Patients with AD and VD were subjected to the Mini‐Mental State Examination (MMSE) before enrollment (Tombaugh & McIntyre, 1992). All participants were subjected to detailed neurological examinations, related laboratory examinations, head computed tomography (CT), and magnetic resonance imaging (MRI; without contraindications). In addition, cerebrospinal fluid was extracted from patients during the physical examination on admission. Written informed consent was signed by all patients, and the study complied with the norms of human research.

The study was approved by the Ethics Committee of The Second Affiliated Hospital of Jiaxing University. All patients or their family members signed written informed consent and follow‐up consent at the time of initial diagnosis.

Inclusion criteria were as follows: (1) MMSE score: illiterate group ≤17 points, elementary school group ≤20 points, and junior high school and above group ≤24 points; (2) Clinical Dementia Rating (CDR) scale ≥1 point; and patients with AD met the DSM‐IV dementia diagnostic criteria and NINCDS‐ADRDA diagnostic criteria. Exclusion criteria were as follows: (1) Patients with other neurological diseases or dementia / cognitive disorders caused by other factors such as poisoning; (2) Patients with severe heart dysfunction and renal dysfunction; and (3) Patients refused to cooperate; thus, the mental state and cognitive state could not be assessed. The inclusion criteria for the Control group were as follows: (1) patients without cognitive dysfunction, no physical dyskinesia, and no alcohol dependence; (2) MMSE score: illiterate group >17 points, elementary school group >20 points, and junior high school and above 24 points; (3) CDR = 0 points, ADL ≤20 points; (4) Hachinski ischemic index ≤4 points; and (5) Nervous system examination was normal. The exclusion criteria for Control group were as follows: (1) Intracranial mass lesion and infection confirmed by CT or MRI; (2) Patients with a history of stroke, head trauma, epilepsy, and multiple sclerosis; (3) Patients with depression, schizophrenia, alcoholism, etc.; and (4) Patients with heart dysfunction, renal dysfunction, and liver dysfunction.

### 2.2 Assessment criteria

Patients were screened by MMSE. Well‐trained professionals with consistent evaluation conducted neuropsychological assessment, including CDR, Activity of Daily Living (ADL), and Neuropsychiatric Inventory (NPI).

Mini‐Mental State Examination scale can be used to screen cognitive defects and assess intelligent state. It contains 30 small items, with a total of score of 30 points. To be specific, items 1–5 are time‐oriented; items 6–10 are location‐oriented; items 11–13 are for language immediate memory; items 14–18 are for computing power and calculation; items 19–21 are for short‐term memory; items 22–23 are for naming; item 24 is for linguistic retelling; item 25 is for reading comprehension; items 26–28 items are for language comprehension; item 29 is for speech expression; and item 30 is for graphic drawing. MMSE scores are closely associated with education level. The cutoff values are as follows: illiteracy group ≤17 points, elementary school group ≤20 points, and junior high school and above groups ≤24 points. Patients below the cutoff value were considered as cognitive impairment or consistent with dementia diagnosis.

#### 2.2.1 CDR scale

This scale is commonly used to assess the degree of dementia at present (Kim, 2014). The assessments include memory, orientation, ability of judging and problem solving, ability of working and social communication, family life and personal hobbies and ability of independent life. Assessment criteria were as follows: CDR 0 point: no dementia, CDR 0.5 point: suspected dementia, CDR 1 point: mild dementia, CDR 2 points: moderate dementia, and CDR 3 points: severe dementia.

#### 2.2.2 ADL

Activity of Daily Living scale includes 14 basic functions necessary for independent life, with the highest score of 64 points. Patients with or less than 24 points indicated normal results. However, ADL scale is not sensitive enough for the diagnosis of dementia, since patients with early‐stage dementia may not have declined life activity.

#### 2.2.3 NPI

A fixed questionnaire is designed according to the pathological and psychiatric symptoms of patients with dementia, which is mainly used to evaluate the psychopathological and other neuropsychiatric disorders of patients with dementia. NPI contains 12 dimensions, including 10 behavioral dimensions (delusion, hallucination, agitation, depression, anxiety, euphoria, indifference, disinhibition, irritability, and behavioral disturbances) and two autonomic functional dimensions (including sleep and nighttime behavior disorders, appetite, and eating disorders).

### 2.3 Protein detection

The cerebrospinal fluid from patients with AD, patients with VD, and healthy controls was centrifuged and subjected to ELISA for detection of GSDMD, T‐Tau, Tau181p, Aβ_1–42_, and inflammatory factors (IL‐1β and IL‐6) according to the manufacturer's instruction. GSDMD ELISA kit was purchased from Abcam (USA, Massachusetts); T‐Tau, Tau181p, and Aβ_1‐42_ ELISA kit was purchased from Invitrogen (Thermo Fisher, USA), and IL‐1β and IL‐6 ELISA kit was purchased from Nanjing Jiancheng Biological Company (Nanjing, China). A microplate reader was used to analyze the protein expression, and the results were shown as pg/ml.

### 2.4 Statistical analysis

Variables with normal distribution were shown as means ± *SD*. Categorical data (such as age and gender) were tested by *χ* ^2^ test. One‐way ANOVA was used for comparison among three groups, and Tukey’s method was used for further comparison in the case of statistical significance. And independent sample *t* test was used for measurement data between two groups. Receiver operating characteristic (ROC) curve was plotted, followed by assessment of the diagnostic and differential diagnostic value of various biomarkers in cerebrospinal fluid via the areas under the ROC curves (AUC). Youden index was used to calculate the optimal diagnostic cutoff point, optimal sensitivity, and specificity of each variable. Youden index was equal to the value of sensitivity minus (1‐specificity). Pearson correlation analysis was further performed. SPSS 20.0 software was used for statistical analysis, and the significance level was set at *p* < .05.

### 2.5 Ethical approval

The study was approved by the Ethics Committee.

## 3 RESULTS

### 3.1 Comparison of clinical data of patients

Gender, age, course of disease, educational level, history of hyperglycemia, history of hypertension, MMSE score, ADL score, NPI score, and CDR score were compared among AD, VD, and Control groups. As a result, there was no significant difference in gender, age, educational level, etc. (*p* < .05). MMSE score and ADL score were not significantly different between patients with AD and patients with VD, while NPI score was significantly higher in patients with VD than patients with AD (*p* < .05; shown in Table 1).

**TABLE 1:** Comparison of clinical data (Means ± *SD* or %)

Variable	AD (*n* = 60)	VD (*n* = 60)	Control (*n* = 50)	*p* Value
Gender (male/female)	28/32	31/29	24/26	.611
Age	63.4 ± 8.4	65.8 ± 9.1	60.7 ± 8.8	.286
Education (years)	8.4 ± 2.3	9.1 ± 1.8	8.5 ± 2.6	.276
Course of disease (month)	63.4 ± 9.8	59.9 ± 10.4	—	.105
History of hyperglycemia	71.7 (43/60)	75.0 (45/60)	76.0 (38/50)	.329
History of hypertension	63.3 (38/60)	66.7 (40/60)	64.0 (32/50)	.597
MMSE	11.5 ± 2.8	12.2 ± 4.8	25.8 ± 3.9	<.001
ADL	35.6 ± 12.5	36.2 ± 12.1	—	.894
NPI	18.3 ± 8.8	28.5 ± 11.5	—	.001
CDR (1/2/3)	12/26/22	11/28/21	—	.581

### 3.2 Comparison of the expression levels of protein marker in cerebrospinal fluid

The levels of GSDMD, T‐Tau, Tau181p, and Aβ_1–42_ in cerebrospinal fluid were significantly different among AD, VD, and Control groups (*p* < .001); meanwhile, the expression level of inflammatory factors (including IL‐1β and IL‐6) was also significantly different among the three groups (*p* < .001). Moreover, in pairwise comparison, the levels of GSDMD, T‐Tau, and Tau181p in cerebrospinal fluid were significantly higher in AD group than those of VD group and Control group (both *p* < .001). The concentration of Aβ_1‐42_ in cerebrospinal fluid was significantly lower in patients with VD and AD than that of healthy controls (*p* < .001), and the level of Aβ_1–42_ was significantly different between patients with AD and patients with VD (*p* < .001). The ratios of GSDMD, T‐Tau, Tau181p, and Aβ_1–42_ in patients with AD were significantly different from those of patients with VD (*p* < .001; shown in Table 2).

**TABLE 2:** Results of expression levels and ratios of protein in cerebrospinal fluid (Means ± *SD*)

Variable	AD (*n* = 60)	VD (*n* = 60)	Control (*n* = 50)	*p* Value
GSDMD (pg/ml)	3.19 ± 0.55	1.35 ± 0.34	0.37 ± 0.08	<.001
T‐Tau (pg/ml)	554.87 ± 65.76	300.40 ± 43.76	216.24 ± 35.65	<.001
Tau181p (pg/ml)	81.37 ± 22.43	42.27 ± 18.65	30.78 ± 11.43	<.001
Aβ_1−42_ (pg/ml)	512.13 ± 43.54	805.77 ± 33.84	912.23 ± 54.87	<.001
IL‐1β (pg/ml)	10.25 ± 2.16	1.95 ± 0.55	0.46 ± 0.11	<.001
IL‐6 (pg/ml)	14.17 ± 3.11	5.43 ± 1.55	2.26 ± 0.88	<.001
GSDMD/T‐Tau (x10^−3^)	5.7 ± 0.08	4.2 ± 0.03	1.7 ± 0.03	<.001
GSDMD/Tau181p (x10^−3^)	4.5 ± 0.03	3.1 ± 0.02	1.2 ± 0.02	<.001
GSDMD/Aβ_1−42_ (x10^−3^)	6.2 ± 0.04	1.6 ± 0.02	0.4 ± 0.01	<.001

### 3.3 ROC curve analysis for GSDMD as diagnostic marker for ad and differential diagnostic marker for VD

**FIGURE 1:**
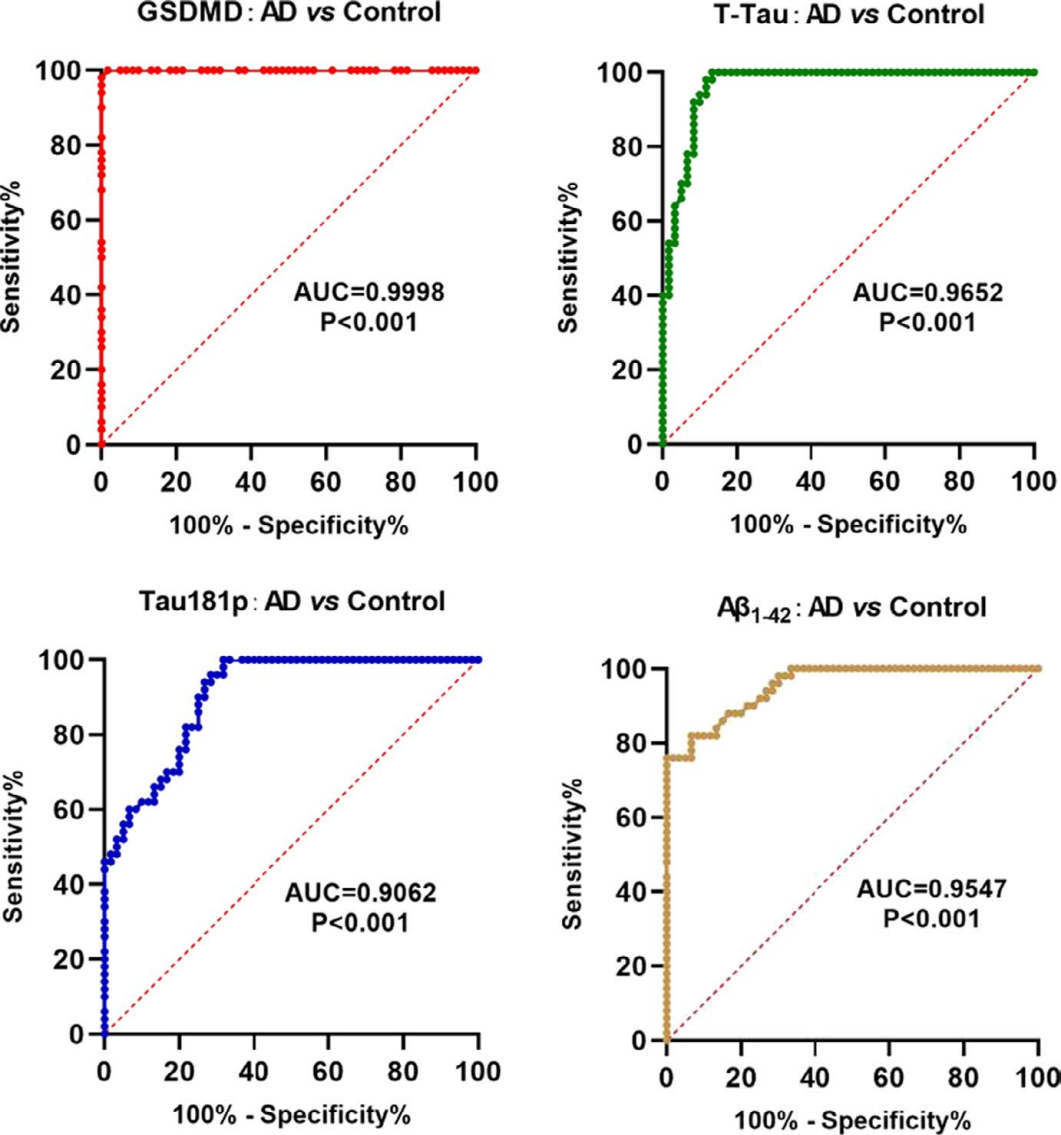
Diagnostic value GSDMD as biomarker

The AUC, optimal cutoff values, sensitivity, and specificity of GSDMD, T‐Tau, Tau181p, and Aβ_1‐42_ in cerebrospinal fluid for AD diagnosis and VD differential diagnosis were shown in Tables 3 and 4. GSDMD in cerebrospinal fluid had a good diagnostic accuracy (AUC = .9998, *p* < .001) for the diagnosis of AD and healthy controls. When GSDMD was at the optimal cutoff value of 3.12, the sensitivity was 92.87 and the specificity was 98.98. In addition, T‐Tau, Tau181p, and Aβ_1–42_ also had good diagnostic value for the diagnosis of AD (AUC = .9652, .9052, .9547, *p* < .001). GSDMD / T‐Tau, GSDMD / Tau181p, and GSDMD / Aβ_1‐42_ also had certain diagnostic value in AD diagnosis (AUC = .822, .767, .798, *p* < .001; shown in Figure 1).

**TABLE 3:** ROC analysis for the diagnosis of AD and healthy controls

Variable	AUC	*p* Value	95% CI		Boundary value (pg/ml)	Sensitivity (%)	Specificity (%)
			Lower bound	Upper bound			
GSDMD	.9998	<.0001	.9991	1.0000	3.12	92.87	98.98
T‐Tau	.9652	<.0001	.9342	.9962	469.87	80.23	91.66
Tau181p	.9062	<.0001	.8233	.9287	84.43	76.98	88.98
Aβ_1−42_	.9547	<.0001	.8763	.9432	532.87	78.98	90.98
GSDMD/T‐Tau	.8225	<.0001	.7873	.8552	2.14	68.98	80.98
GSDMD/Tau181p	.7677	<.0001	.7111	.8334	2.14	69.43	82.76
GSDMD/Aβ_1−42_	.7982	<.0001	.7663	.8985	3.11	72.88	85.77

**TABLE 4:** ROC analysis for the differential diagnosis between AD and VD

Variable	AUC	*p* Value	95% CI		Boundary value (pg/ml)	Sensitivity (%)	Specificity (%)
			Lower bound	Upper bound			
GSDMD	.8765	<.0001	.8131	.9490	2.88	82.87	80.88
T‐Tau	.7894	<.0001	.7081	.8708	522.43	71.88	72.76
Tau181p	.8234	<.0001	.7433	.8911	92.87	78.99	89.13
Aβ_1−42_	.7676	<.0001	.6843	.8549	623.98	71.98	69.88
GSDMD/T‐Tau	.7122	<.0001	.606	.8015	2.27	61.87	58.99
GSDMD/Tau181p	.7411	<.0001	.611	.794	2.43	60.44	59.39
GSDMD/Aβ_1−42_	.7233	<.0001	.732	.849	3.43	65.87	62.54

**FIGURE 2:**
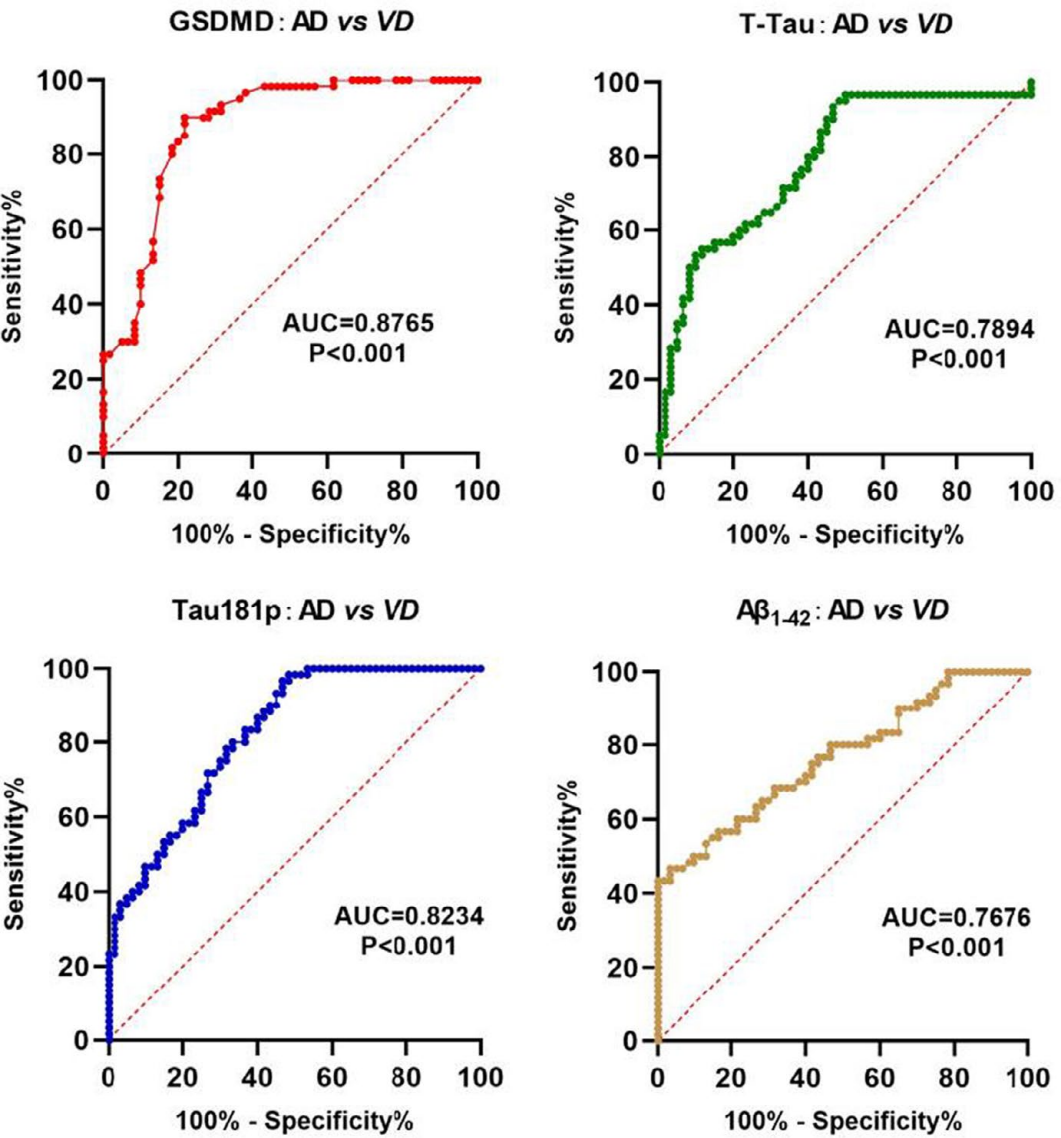
Differential diagnostic value of GSDMD between AD and VD

In the differential diagnosis of AD and VD, GSDMD in cerebrospinal fluid had a good diagnostic accuracy (AUC = .8765, *p* < .001). When GSDMD was at the optimal cutoff value of 2.88, the sensitivity was 82.87 and the specificity was 80.88. Moreover, T‐Tau, Tau181p, and Aβ_1–42_ also had good diagnostic value for the diagnosis of AD (AUC = .7894, .8234, .7676, *p* < .001). GSDMD / T‐Tau, GSDMD / Tau181p, and GSDMD / Aβ_1‐42_ also had certain diagnostic value in AD diagnosis (AUC = .7122, .7411, .7233, *p* < .001; shown in Figure 2).

### 3.4 Correlation analysis

Correlation analysis showed that the levels of GSDMD, T‐Tau, Tau181p, and Aβ_1–42_ in cerebrospinal fluid were not significantly correlated with gender, age, and course of disease (*p* > .05), which were also not significantly associated with the degree of cognitive impairment in MMSE, CDR, ADL, and NPI scales (*p* > .05).

**FIGURE 3:**
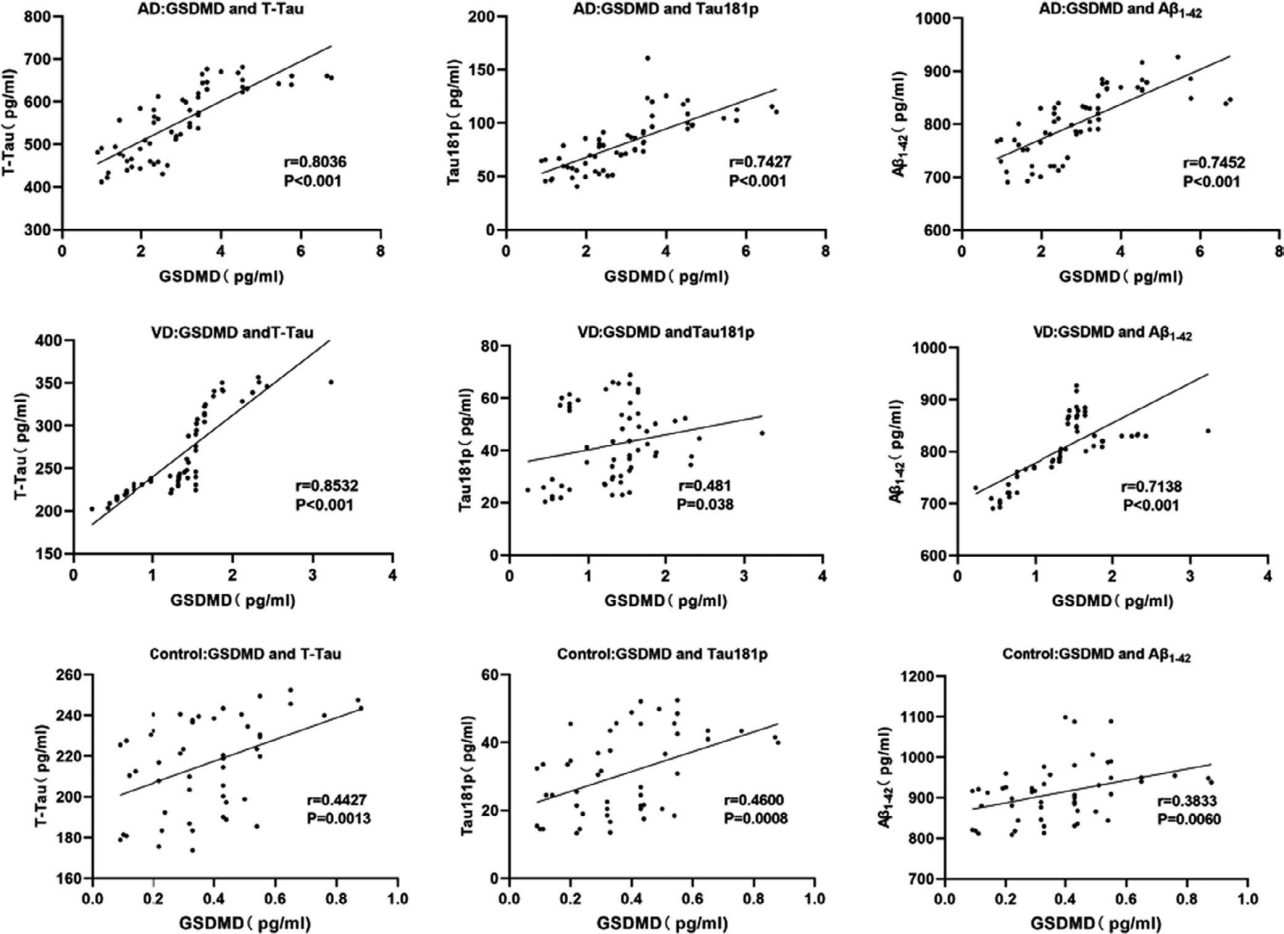
Correlation of biomarkers in cerebrospinal fluid

GSDMD level in cerebrospinal fluid was correlated with the levels of T‐Tau, Tau181p, and Aβ_1‐42_ (*r* = .8036, .7472, .7452, *p* < .001) in patients with AD, GSDMD level was correlated with the levels of T‐Tau, Tau181p, and Aβ_1‐42_ in patients with VD (*r* = .8352, .4810, .713, *p* < .001, *p* = .038, *p* < .001, respectively), and GSDMD level was correlated with the levels of T‐Tau, Tau181p, and Aβ_1‐42_ in healthy controls (*r* = .4427, .4600, .3833, *p* = .0013, .0008, .0060, respectively; shown in Table 5 and Figure 3).

**TABLE 5:** Correlation analysis of biomarkers in cerebrospinal fluid (*r*)

Index	*R* value	*p* Value
AD group		
GSDMD and T‐Tau	.8036	<.001
GSDMD and Tau181p	.7472	<.001
GSDMD and Aβ_1−42_	.7452	<.001
VD group		
GSDMD and T‐Tau	.8532	<.001
GSDMD and Tau181p	.4810	.038
GSDMD and Aβ_1−42_	.7138	<.001
Control group		
GSDMD and T‐Tau	.4427	.0013
GSDMD and Tau181p	.4600	.0008
GSDMD and Aβ_1−42_	.3833	.0060

### 3.5 Correlation between inflammatory factors and the expression of protein marker in cerebrospinal fluid of patients with AD

**FIGURE 4:**
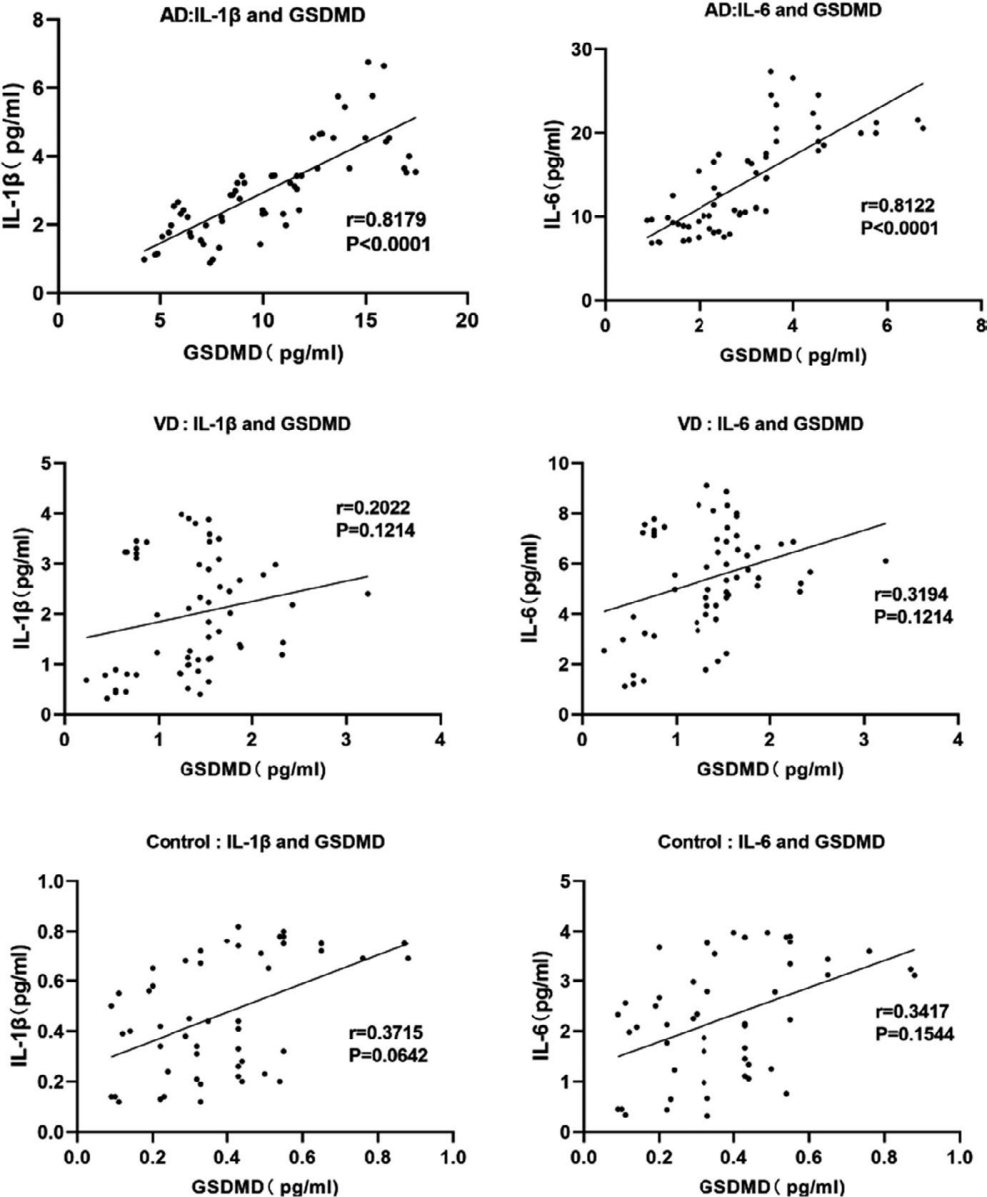
Correlation between inflammatory factors and the expression of protein marker in cerebrospinal fluid of patients with AD

Correlation analysis showed that the levels of IL‐1β and IL‐6 in the cerebrospinal fluid were correlated with GSDMD expression in patients with AD (*r* = .8197, .8122, *p* < .0001). However, the levels of IL‐1β and IL‐6 were not significantly correlated with GSDMD expression in cerebrospinal fluid among patients with VD and healthy controls (VD: *r* = .2022, .3194, *p* = .1214, .1322, respectively; Control: *r* = .3715, .3417, *p* = .0642, .1544, respectively). The above results showed that GSDMD expression was only correlated with the levels of IL‐1β and IL‐6 in the cerebrospinal fluid of patients with AD (shown in Table 6 and Figure 4).

**TABLE 6:** Correlation between inflammatory factors and the expression of protein marker in cerebrospinal fluid of patients with AD (*r*)

Index	*R* value	*p* Value
AD group		
IL‐1β and GSDMD	.8179	<.001
IL‐6 and GSDMD	.8122	<.001
VD group		
IL‐1β and GSDMD	.2022	.1214
IL‐6 and GSDMD	.3194	.1322
Control group		
IL‐1β and GSDMD	.3715	.0642
IL‐6 and GSDMD	.3417	.1544

## 4 DISCUSSION

Since the first case report of AD by Alois Alzheimer in 1906, the pathogenesis of AD is correlated with Aβ deposition, NFT of Tau protein and neuroinflammation after decades of research (Mo et al., 2017; Shimada et al., 2017). Recent studies have revealed that neurovascular injury, oxidative stress injury, etc. also play important roles in AD (Popp et al., 2017; Valero et al., 2017). At present, the diagnostic guidelines have been proposed by NIA‐AA and IWG, which clearly demonstrate that Aβ is decreased and Tau protein is increased in cerebrospinal fluid; meanwhile, Aβ deposition and AD susceptibility gene mutations could be used for staging and classification of AD indicated by PET images (Prestia et al., 2015). However, the sensitivity and specificity of the diagnostic criteria are different, which varies greatly among individuals.

In recent years, studies have found that new types of markers, such as neurofilament protein (NFL), VirE2 interacting protein 1 (VLP‐1), hippocampal neurogranin (Ng), and nuclear synaptic protein, which can be potentially applied for AD diagnosis (Hampel et al., 2018).

Neuroinflammation is one of the main pathological mechanisms of AD. Aβ deposition activates different cell receptors and intracellular signals. Microglia can transcribe into inflammatory cytokines, reactive oxygen species, NADPH oxidase (NOX), etc. Several markers have also been reported in the inflammatory response. YKL‐40 is a secretory glycoprotein and is associated with various diseases. Meanwhile, studies on AD have shown that YKL‐40 level is increased in cerebrospinal fluid (Muszyński et al., 2017). Monocyte chemokine protein (MCP‐1) has the strongest inflammatory activity. The plasma level of MCP‐1 is increased in AD and MCI patients, which is associated with cognitive ability (Kai et al., 2018). Moreover, the inflammatory factors, such as TNF‐α, IL‐1β, and IL‐6, have certain diagnostic value. In recent years, pyroptosis has been widely investigated in neurological diseases. Pyroptosis is a type of inflammatory cell death, characterized by swelling of cell membranes and massive expression and release of cytokines. GSDMD is the executive protein of pyroptosis. In the early stage of inflammation, GSDMD is massively expressed, cleaved into p30‐GSDMD by caspase family, thereby opening cell membrane pores; therefore, GSDMD is a marker of pyroptosis. At present, pyroptosis has been found to be involved in AD, while its diagnostic value remains unknown. VD is a cognitive disorder caused by vascular disease, generally as a secondary disease. At present, the etiology of VD has not been revealed. Due to the significant cognitive impairment in both AD and VD in clinical practice, it is difficult to timely and accurately diagnose and differentiate AD and VD (Vishnu et al., 2017). At present, there is no specific role of pyroptosis in VD; thus, we speculate that protein marker of pyroptosis is of diagnostic value.

In this study, ELISA was used to detect the expression of GSDMD in cerebrospinal fluid; meanwhile, the classic markers T‐Tau, Tau181p, and Aβ_1–42_ were used as controls. As a result, the expression of GSDMD in cerebrospinal fluid of patients with AD and VD was significantly increased, while the levels of T‐Tau and Tau181p were also increased, and the level of Aβ_1–42_ was down‐regulated, which was consistent with the previous reports. The increased expression of GSDMD indicates that pyroptosis plays a certain role in AD, which is associated with the release of inflammatory factors. Further detection showed that the levels of IL‐1β and IL‐6 were also increased in AD. GSDMD and IL‐1β can be cleaved by caspase (Schneider et al., 2017). The expression of both was increased, which was consistent with expectations. ROC analysis revealed that the diagnostic value of GSDMD (AUC = .8765) was superior than the AUC values of the classic markers, including T‐Tau, Tau181p, and Aβ_1‐42_, along with good specificity and sensitivity. Correlation analysis showed that GSDMD expression in cerebrospinal fluid of patients with AD was correlated with T‐Tau, Tau181p, and Aβ_1–42_. However, GSDMD expression in the cerebrospinal fluid was correlated with the levels of IL‐1β and IL‐6 only in patients with AD, but not in patients with VD or healthy controls. The above outcomes indicate that pyroptosis only exists in patients with AD, but not in patients with VD. The high correlation of GSDMD expression with the levels of IL‐1β and IL‐6 also confirmed the role of pyroptosis in AD. In terms of differential diagnosis with VD, GSDMD was superior than T‐Tau, Tau181p, and Aβ_1–42_.

Taken together, in this study, we found that the expression of GSDMD, an executive protein of pyroptosis, was increased in cerebrospinal fluid of patients with AD, which was of certain diagnostic value of AD and differential diagnostic value of VD. Moreover, according to the present data, GSDMD is superior to traditional biomarkers. However, more cases are warranted for further validation.

## CONFLICT OF INTEREST

We have no conflicts of interest to declare.

### PEER REVIEW

The peer review history for this article is available at https://publons.com/publon/10.1002/brb3.2063.

## Data Availability

The data that support the findings of this study are available from the corresponding author upon reasonable request.
